# Chlorotoxin-functionalized mesoporous silica nanoparticles for pH-responsive paclitaxel delivery to Glioblastoma multiforme

**DOI:** 10.1016/j.heliyon.2024.e41151

**Published:** 2024-12-14

**Authors:** Mirjana Mundžić, Amelia Ultimo, Minja Mladenović, Aleksandra Pavlović, Oliviero L. Gobbo, Eduardo Ruiz-Hernandez, Maria Jose Santos-Martinez, Nikola Ž. Knežević

**Affiliations:** a BioSense Institute, University of Novi Sad, Dr Zorana Djindjica 1, 21000, Novi Sad, Serbia; b School of Pharmacy and Pharmaceutical Sciences, Panoz Institute, Trinity College Dublin, D02PN40, Dublin, Ireland; c Trinity St. James's Cancer Institute, St James's Hospital, D08 NHY1, Dublin, Ireland; d School of Medicine, Trinity College Dublin, D02 E8C0, Dublin, Ireland

**Keywords:** Mesoporous silica nanoparticles, Paclitaxel, Glioblastoma multiforme, Targeted drug delivery, pH-responsive, Cyclodextrin, Chlorotoxin

## Abstract

Glioblastoma multiforme (GBM) is a highly aggressive brain cancer associated with poor survival rates. We developed novel mesoporous silica nanoparticles (MSNs)-based nanocarriers for pH-responsive delivery of a therapeutic drug Paclitaxel (PTX) to GBM tumor cells. The pores of MSNs are loaded with PTX, which is retained by β-cyclodextrin (CD) moieties covalently linked to the pore entrances through a hydrazone linkage, which is cleavable in weakly acidic environment. Furthermore, we utilized a host-guest interaction between the adamantane and capping CD moieties to further functionalize the surface with a potential glioma-targeting oligopeptide chlorotoxin (CHX). *In vitro* studies in the U87 GBM cell line show decreased uptake, but increased toxicity of CHX-modified nanoparticles compared to CHX-free nanoparticles. The obtained results are promising toward development of advanced drug nanocarriers, which may target the overexpressed receptors in cancer tissues and utilize their weakly acidic environment for triggering the drug release, potentially leading to more efficient cancer treatments.

## Highlights

• Successfully developed pH-responsive MSNs for PTX delivery to GBM cells.
• The acidic environment triggers PTX release - promising for GBM treatment.
• PTX-loaded MSNs show higher cytotoxicity when functionalized with CHX.

## Graphical abstract

**Figure:**
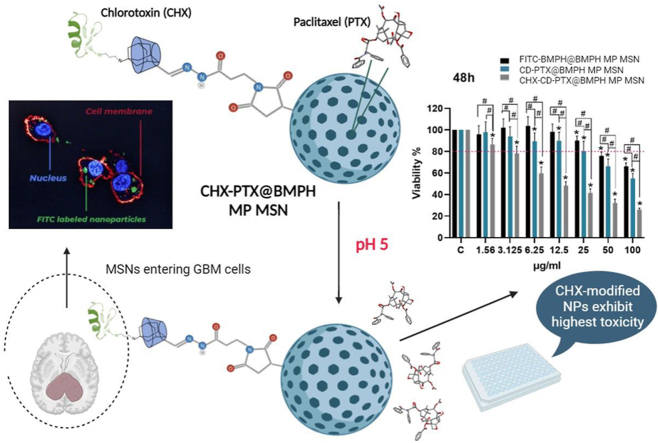

## 1 Introduction

Stimuli-responsive drug delivery systems (DDSs) have significant potential for targeted drug delivery, mitigating the harmful effects of cytotoxic drugs. With the remarkable advancements in nanotechnology, nanomaterials have been integrated into the biomedical field, leading to the development of numerous nanoparticles (NPs) as drug delivery systems for both diagnostic and therapeutic purposes. Mesoporous silica nanoparticles (MSNs) are excellent candidates for constructing "smart" drug carriers [1]. These materials possess an exceptionally large specific surface area, high porosity, particle sizes ranging from 100 to 200 nm, and surface modification possibilities. All these offer numerous opportunities for designing nanotherapeutics for targeted tumor therapy in response to the tumor environment [2]. Selective functionalization of MSNs involves various modifications on the external particle surface as well as within the pores. Nanosystems serving as drug carriers can be designed to respond, i.e., release the drug, upon environmental changes, such as alterations in pH values [3], the presence of specific biomolecules [4], exposure to light [5] or other external stimuli such as ultrasound [6]. This is achieved using different agents that block mesopores and entrap the drugs, regulating the release kinetics of the drug trapped within the mesopores in nanosystems and thereby modulating their activity [[7], [8], [9]].

Nanoparticles demonstrate passive targeting ability towards tumor tissue due to the enhanced permeability and retention effect (EPR), wherein nanoparticles of specific size and surface characteristics spontaneously accumulate in the tumor microenvironment [10]. External surface functionalization of nanoparticles with suitable biomolecules (e.g., proteins, antibodies, folic acid, sugars) can also be utilized for additional, active targeting of drug delivery to tumor tissues [11].

Glioblastoma multiforme (GBM) is one of the most common and aggressive types of brain tumors. Lack of nutrients, oxidative stress, low oxygen levels, and high glucose adjust cancer cells to rely on anaerobic glycolysis. This process leads to a significant accumulation of lactic acid, which is then expelled into the extracellular environment, resulting in elevated proton concentration [12]. Because of that, research from multiple studies has revealed that tumors can exhibit weakly acidic extracellular environment [13]. In the case of GBM, John et al. [14] presented a diagram that maps the different zones of the tumor and their corresponding pH levels, highlighting the distinct pH microenvironments within each zone and revealing low pH zones from 5.5 down to as low as 3.4.

The primary treatment for GBM involves removing as much of the tumor as possible, followed by radiation therapy and oral temozolomide (TMZ) administration. However, this approach typically only increases life expectancy of patients by 16–18 months [15]. Removing all tumor tissue during surgery, especially brain tissue, is known to be dangerous and impractical, and prolonged radiotherapy and chemotherapy treatments often lead to significant resistance. Furthermore, glioblastoma high phenotypic and genotypic diversity contributes to multidrug resistance and complicates targeted drug delivery. The effectiveness and bioavailability of therapeutic drugs are largely hindered by the tumor microenvironment, stem cells, immune response, and particularly the blood–brain barrier (BBB) [16]. Recent studies have shown that chlorotoxin (CHX), a 36-amino acid neurotoxin isolated from scorpion venom, targets voltage-gated chloride channels, including the calcium-dependent phospholipid-binding protein annexin-2 and the inducible extracellular enzyme matrix metalloproteinase (MMP)-2 [[17], [18], [19]]. Notably, MMP-2 is typically over-expressed in GBM compared to normal brain cells and plays an important role in physiological processes such as angiogenesis, metastasis and cellular invasion [20]. Moreover, recent reports have clearly demonstrated that CHX can be utilized for helping NPs to cross the BBB and for effective imaging and therapy of brain cancers [19,[21], [22], [23]]. Consequently, with the right design, this approach holds promise for using MSNs to transport drugs into the brain through transcellular transport [24]. Previous *in vivo* experiments have proven that MSNs exhibit biocompatibility, have preference for accumulating within tumor sites and efficiently transport drugs to cancerous tissues [25].

**Scheme 1:**
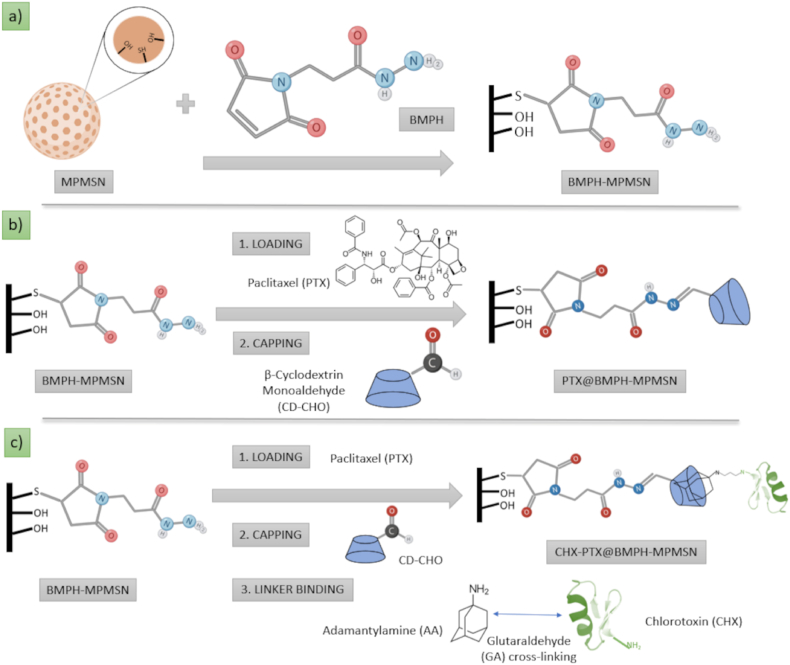
a) Formation of N-β-maleimidopropionic acid hydrazide-mercaptopropyl MSN (BMPH-MPMSN). b) Loading of anticancer drug paclitaxel (PTX) and pore capping with β-cyclodextrin monoaldehyde (CD-CHO). c) Loading of PTX, pore capping with CD-CHO and attachment of chlorotoxin (CHX).

In the current study we loaded the pores of MSNs with paclitaxel (PTX), a widely used anticancer drug, and entrapped by pore-blocking with CD monoaldehyde (CD-CHO) (Scheme 1). Paclitaxel (PTX) is known for its activity against cancer by stabilizing microtubules [26], though its full potential for the treatment of brain cancers has been hindered by the protective nature of the BBB [27]. Recently, Zhang et al. have shown that formulation of PTX with albumin becomes a potent treatment for glioma upon ultrasound-assisted disruption of the BBB [28], while a synergistic activity of albumin-bound PTX in combination with temozolomide against GBM was also demonstrated [29]. Therefore, we opted for using the CHX-functionalized MSN as a nanocarrier for PTX, with an added benefit of having CDs as pore-blocking moieties. Namely, CDs are typically used in the pharmaceutical industry as complexing agents to increase the aqueous solubility of poorly soluble drugs [30]. Hence, their use may be helpful for improving the solubilization of the poorly soluble PTX if jointly released from the NPs, though their primary function in our NP assembly was as gatekeepers [31,32]. Functionalization of the nanoparticles with oligopeptide CHX was achieved by covalently attaching the peptide to adamantane-amine (AA), followed by the host-guest interaction between AA and the pore-capping CD moieties (Scheme 1c).

Furthermore, the materials were constructed with an acidification-responsive hydrazone linker between the MSN and the pore-capping CDs, for triggering the drug delivery in response to acidic environment of tumor tissues as well as endolysosomes [33]. The starting material N-β-maleimidopropionic acid hydrazide-mercaptopropyl MSN (BMPH-MPMSN) from the current study was shown applicable for construction of acidification-responsive nanocarriers of contrast agents for magnetic resonance imaging (MRI) and for MRI-based tracking of the cargo release process in our recent study [34]. As detailed therein, we aimed to showcase the applicability of MRI to signal the successful drug delivery to desired tissues. Once such confirmation is obtained by MRI, the same nanocarrier filled with anticancer drugs would be used for the treatment. Hence, we report herein a follow-up study with the same starting material (BMPH-MPMSN) used for loading and acidification-responsive release of anticancer drug PTX from the CD-capped BMPH-MSN, as well as for covalent attachment of CHX as a potential targeting moiety. The expected mechanism for acidification-responsive PTX release is well-known cleavage of hydrazone linkage upon acidification, which leads to removal of the pore-capping cyclodextrin and allowing PTX to diffuse from the mesopores. The release kinetics of PTX from the materials were measured at different pH levels using UV spectrophotometry, while the capabilities for cancer treatment were evaluated by PTX-loaded materials on GBM cell viability *in vitro*. The additional materials without the loaded PTX and labeled with fluorescein isothiocyanate (FITC) were also prepared to analyze the uptake of nanomaterials by GBM cells through confocal microscopy and flow cytometry.

## 2 Materials and methods

All chemicals were used directly as purchased. Paclitaxel (PTX) was bought from Alfa Aesar. Tetraethoxysilane (TEOS), 3-aminopropyltrimethoxysilane (APTMS), 3-mercaptopropyl trimethoxysilane (MPTMS), cetyltrimethylammonium bromide (CTAB), β-cyclodextrin (CD), Dess Martin periodinane (DMP), adamantylamine hydrochloride (AA), N-β-maleimidopropionic acid hydrazide, trifluoroacetic acid salt (BMPH), glutaraldehyde (GA), chlorotoxin (CHX), Fluorescein Isothiocyanate (FITC), dimethyl sulfoxide (DMSO), and dichloromethane (DCM) were purchased from Sigma-Aldrich (St. Louis, MA, USA). Minimum Essential Medium Eagle (MEM), L-glutamine, fetal bovine serum (FBS), and Hoechst 33342 were bought from Merck KGaA (Darmstadt, Germany). The ActinRed™ 555 ReadyProbes™ Reagent was provided by Thermo Fisher Scientific (Waltham, MA, USA), and the Cell Proliferation/Cytotoxicity Assay CCK-8 was obtained from Dojindo EU GmbH (Munich, Germany).

The U87 cell line (Catalogue Number: HTB-14, RRID: CVCL 0022) was authenticated upon receipt from ATCC and periodically during the study. Authentication included STR profiling to confirm genetic identity, morphological evaluation to verify cell characteristics, and growth pattern assessment to match documented profiles. The cell line was also tested for mycoplasma contamination using PCR, fluorescence microscopy, and biochemical assays. All tests confirmed the cell line was free of contamination.

### 2.1 Synthesis of MPMSN and BMPH-MPMSN

The synthesis of MPMSN and functionalization with BMPH to obtain the starting material (BMPH-MPMSN) for loading the anticancer drug PTX was performed as recently reported in our study [34].

Briefly, a solution of NaOH (aq) (2 M, 3.50 mL) was added to a suspension of cetyltrimethylammonium bromide (CTAB, 1 g, 2.74 mmol) in 480 mL of water, followed by adjusting the temperature to 80 °C. Tetraethyl orthosilicate (TEOS, 5 mL, 25.7 mmol) was first introduced dropwise to the surfactant solution, followed by dropwise addition of (3-mercaptopropyl) trimethoxysilane (MPTMS, 0.97 mL, 5.13 mmol). The mixture was allowed to stir for 2 h to give rise to a white precipitate (as-synthesized MPMSN). The solid product was filtered, washed with copious amounts of deionized water and ethanol, and dried at 80 °C. Final mercaptopropyl-MSN material (MPMSN) was obtained upon removal of the CTAB surfactant template by extraction with a solution of concentrated HCl in methanol (1 %, v/v). After 6 h of extraction the material was additionally washed with deionized water and ethanol, and again dried at 80 °C.

Synthesis of BMPH-MPMSN (Scheme 1a): 120 mg of BMPH were dissolved in 40 ml of water followed by the addition of 800 mg of MPMSN. The mixture was stirred for 24h at room temperature. The material was isolated via centrifugation, washed three times with water, once with ethanol and dried.

### 2.2 Synthesis of FITC-labeled materials

Fluorescein isothiocyanate (FITC, 1 mg, 2.6 μmol) was added to APTMS (2.6 μmol) in dry DMSO (0.5 mL), stirred for 30 min and then added to a suspension of BMPH-MPMSN (25 mg) in 10 mL of dry toluene. The suspension was refluxed for 6 h at 110 °C, under nitrogen. After cooling, the resulting material was centrifuged, washed with toluene and ethanol. The obtained FITC-BMPH-MPMSN material was centrifuged and dried at 80 °C.

Procedure for preparing empty (not loaded with PTX), CD capped FITC-labeled materials: solution of CD-CHO (20 mg), previously prepared as reported in Ref. [34]., in 2 mL of PBS was added to 20 mg of FITC-BMPH-MPMSN and stirred for 24 h at room temperature. The mixture was centrifuged for 10 min at 11000 rpm, the supernatant decanted and a solution of AA (0.06 mmol, 11.4 mg) in 1 mL of phosphate buffer (0.1 M) was added to the precipitate. After stirring at room temperature for 16 h and washing with PBS, the material was resuspended in 10 mL of PBS and divided into two equal fractions. One of the fractions was centrifuged and dried at 80 °C to yield CD-FITC-BMPH-MPMSN material. The second sample was centrifuged and a solution of 0.1 mmol of GA dissolved in 1 mL of PBS was added to the material. The mixture was allowed to stir at room temperature for 2 h and then washed three times with PBS. The material was then redispersed in 1 mL of PBS and 0.1 mL of CHX (4 μg) aqueous solution was added. The stirring continued for 24 h at room temperature in the dark, the material was washed three times with PBS and dried in air to yield the empty FITC-labeled material, capped with CD and attached CHX (CHX-CD-FITC-BMPH-MPMSN).

### 2.3 Synthesis of PTX@BMPH-MPMSN and CHX-PTX@BMPH-MPMSN

Anticancer drug PTX (14.07 mg) was dissolved in 3 mL of DCM. To this solution 150 mg of BMPH-MPMSN was added and the mixture was allowed to stir for 24 h at room temperature in the dark. The DCM was slowly evaporated, 3 mL of PBS solution (10 mM, pH 7.4) containing 150 mg of CD-CHO was added and the mixture was stirred for another 20 h at room temperature in the dark. The reaction mixture was divided into two fractions and one half was centrifuged, washed three times with 15 mL of PBS and twice with 2 mL of acetonitrile. Finally, the obtained PTX@BMPH-MPMSN material was dried in air.

The second fraction was further modified with glutaraldehyde-adamantylamine (GA-AA) conjugate, which was first prepared by mixing 0.03 mmol GA and 0.03 mmol AA in PBS solution (1 mg/mL). After addition of GA-AA, the stirring continued for 6 h under the same conditions. The material was then centrifuged, washed twice with PBS, and redispersed in 1 mL of water containing 250 μL of CHX solution (40 μg/mL). The stirring continued for 24 h in the dark at room temperature and the as-synthesized CHX-PTX@BMPH-MPMSN material was isolated by centrifugation, washed with PBS, and dried in air.

### 2.4 Scanning electron microscopy (SEM)

The morphological examination of the powders was carried out on Apreo 2 C Scanning Electron Microscope (SEM, Thermo Fisher Scientific, Waltham, MA, USA). Samples were placed on copper (Cu) tape to lessen the effects of charging. The images were taken at high vacuum with the SEM operating at 1 kV to 500V and 25–100 pA at a NaOH (aq) (2 M, 3.50 mL) was added.

### 2.5 Transmission electron microscopy (TEM)

TEM analysis was carried out using an FEI Talos F200X microscope operating at 200 keV in standard mode. The samples were prepared by dispersing the nanoparticles in ethanol, placing a drop of this mixture onto a carbon-coated copper grid, and letting it dry naturally in the air.

### 2.6 Thermogravimetric analyses (TGA) an*d* differential scanning calorimetry (DSC)

The TGA and DSC analyses were carried out using a Netzsch STA 449 F5 Jupiter instrument. Approximately 5 mg of each sample was analyzed, under a nitrogen atmosphere, starting from 20 to 800 °C.

### 2.7 Fourier Transform infrared spectroscopy (FTIR)

FTIR spectra were obtained using a Jasco FT/IR-6600 spectrometer. The measurements were performed in attenuated total reflectance (ATR) mode, which allows for direct analysis of solid samples without the need for extensive sample preparation. The solid samples were placed in direct contact with the ATR crystal, ensuring optimal interaction with the infrared beam.

### 2.8 Quantification of the loaded PTX anticancer drug

The amount of loaded PTX was calculated by UV spectroscopy on a Jasco V-750 instrument, by measuring the absorbance values at 230 nm from the mixtures of methanol (65.8 %) and aqueous solutions, by adjusting a published procedure [35]. Calibration curves of PTX were obtained in solvent mixtures containing 65.8 % of methanol and 34.2 % of the following: acetate buffer at pH 5, PBS at pH 6 or pH 7.4, or water. The acetonitrile supernatant was previously evaporated, while dry residue was then dissolved in water/methanol solution in order to measure the PTX concentration by UV spectrophotometry. The formulae used to calculate PTX concentration are: A = 0.0291c + 0.0303 (pH 5/methanol (65.8 %)), A = 0.0322c + 0.0472 (pH 6/methanol (65.8 %)), A = 0.0314c + 0.0466 (pH 7.4/methanol (65.8 %)) and A = 0.0291c + 0.0303 (water/methanol (65.8 %)), where A is the absorbance of the sample at 230 nm.

### 2.9 Measurements of release kinetics of PTX

The release of PTX was performed from a suspension of materials (PTX@BMPH-MPMSN and CHX-PTX@BMPH-MPMSN) in solvent mixtures containing 65.8 % methanol and 34.2 % of acetate buffer at pH 5 or PBS at pH 7.4. At predetermined time points, the suspensions were centrifuged, and the supernatants were analyzed by UV measurements to determine the amount of released PTX based on the absorbance peak at 230 nm, followed by returning the sample to the stirred suspension.

### 2.10 Nitrogen adsorption-desorption measurements

Anton Paar NOVAtouch LX2 instrument was used for the analyses after degassing the materials for 6 h at 105 °C under vacuum. The Brunauer–Emmett–Teller (BET) specific surface area of the materials was calculated from the adsorption branch of the isotherm, specifically in the relative pressure range from 0.05 to 0.3 P/P°. Additionally, Barrett, Joyner, and Halenda (BJH) calculations for pore diameter were conducted using the desorption branch of the isotherm.

### 2.11 Dynamic light scattering measurements (DLS)

DLS and zeta potential measurements of the synthesized nanoparticles were conducted using a Particle size analyzer—Litesizer 500 (Anton Paar, Graz, Austria). To determine the hydrodynamic diameter of the materials, measurements were carried out on suspensions of the materials in water at a final concentration of 0.1 mg/mL, and the analysis was repeated five times.

For investigating the stability of CHX-CD-BMPH-MPMSN and CD-BMPH-MSN materials, the materials were prepared with the same procedure as for FITC-labeled capped materials, starting with BMPH-MPMSN. The measurements were performed in triplicate at concentration of 0.1 mg/mL in cell medium, while the stability of PTX@BMPH-MPMSN and CHX-PTX@BMPH-MPMSN was evaluated in PBS at concentration of 0.05 mg/mL. Measurements were taken immediately after 30 min of sonication and after 1 h of incubation at room temperature without agitation.

### 2.12 Testing materials on glioblastoma cells

U87 cells (human glioblastoma cell line, acquired from the American Type Culture Collection, Catalogue Number: HTB-14, RRID: CVCL 0022) were used to assess the developed nanosystems *in vitro*. The cells were cultured at 37 °C in an atmosphere of 5 % carbon dioxide and 95 % air, in MEM containing 1 % L-glutamine, phenol red and 10 % fetal bovine serum. Cells were split twice a week.

### 2.13 Cellular uptake experiments

All FITC-labeled materials (FITC-BMPH-MPMSN, CD-FITC-BMPH-MPMSN and CHX-CD-FITC-BMPH-MPMSN) were incubated with U87 cells to study NPs internalization by both confocal microscopy and flow cytometry. To analyze NPs internalization by confocal microscopy, U87 cells were seeded at a concentration of 35,000 cells/well in duplicates in IBIDI μ-slide 8 well chamber slides, incubated overnight and treated with the different sets of NPs at a concentration of 50 μg/mL during 2, 4 and 6 h. Then, the cells were washed twice with PBS, fixed with 4 % formaldehyde for 20 min, washed again with PBS and stained. Hoechst 33258 (1 μg/mL) was used to stain the nuclei, while ActinRed™ 555 ReadyProbes™ Reagent by Invitrogen (2 drops/mL) was used to stain cell membranes. After further washes with PBS, the cells were observed with a Leica SP8 laser confocal microscope (Leica Microsystems, Wetzlar, Germany).

For flow cytometry experiments, U87 cells were seeded at a concentration of 300,000 cells/well in 6-well plates (by duplicate), incubated overnight and treated with 50 μg/mL NPs during 2, 4 and 6 h. Then, the cells were washed with media and PBS, detached with trypsin, centrifuged, and fixed with 4 % formaldehyde for 20 min. After centrifugation, the cells were resuspended in PBS and the cell-associated fluorescence (CAF = % positive cells ∗ mean fluorescence intensity) was measured by flow cytometry using a BD Accuri™ C6 Flow Cytometer (BD Biosciences, Franklin Lakes, NJ, USA).

### 2.14 Cell viability assays

The effects of the nanomaterials on cell viability were also studied in U87 cells. For this purpose, 4000 cells/well were seeded in 96-well plates in triplicates, incubated overnight and then treated with a range of NPs concentrations (from 1.56 to 100 μg/mL) and further incubated during 24, 48 and 72 h. Cell viability was then measured using the Cell Proliferation/Cytotoxicity Assay CCK-8 by Dojindo, following the supplier's instructions. The absorbance in the plates was measured at 450 nm using a BioTek Synergy H1 microplate reader (Agilent, Santa Clara, CA, USA).

### 2.15 Statistical analysis

For cell assays, three independent experiments were carried out in duplicate or triplicate as stated. Data were analyzed using GraphPad Prism 8 software (GraphPad Software, La Jolla, CA, USA). All means are reported with standard deviation. Two-way analysis of variance (ANOVA) followed by Sidak's multiple comparisons test were performed as appropriate. Statistical significance was considered at p < 0.05.

## 3 Results and discussion

We previously reported the application of the starting BMPH-MPMSN material for entrapping contrast agents for magnetic resonance imaging (MRI) [34], which demonstrated capabilities for MRI-based tracking of the acidification-responsive cargo release. As described, the general idea is that MRI could be applied in the future to first detect the nanoparticles that are capable of delivering the loaded cargo to a desired tissue, and these identified nanoparticles would then be utilized for delivering the therapeutic molecules to the same tissue. Therefore, as a follow-up proof-of-concept study, we herein characterize the capabilities of the same starting BMPH-MPMSN material for loading anticancer drug PTX, its entrapment by covalent binding of CD (Scheme 1b), attachment of a potential cancer targeting ligand-CHX (Scheme 1c), along with *in vitro* investigation of cell uptake and acidification-responsive drug delivery to Glioblastoma multiforme.

**Fig. 1:**
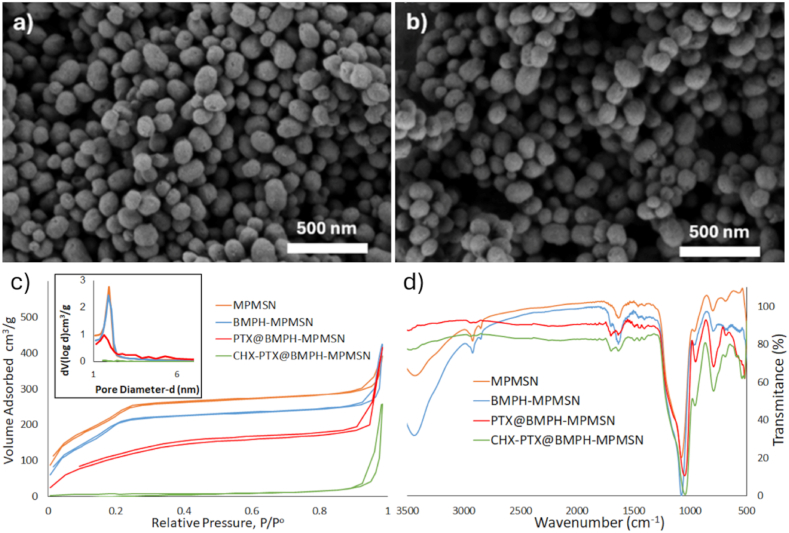
a) SEM image of PTX@BMPH-MPMSN, b) SEM image of CHX-PTX@BMPH-MPMSN, c) nitrogen sorption isotherms (inset shows BJH pore size distribution) and d) FTIR spectra of PTX@BMPH-MPMSN and CHX-PTX@BMPH-MPMSN. The results for the newly constructed materials are compared to the characteristics of the starting MPMSN and BMPH-MPMSN, which are reproduced with permission from Ref. [34].

The SEM images of drug-loaded and pore-capped nanoparticles show spherical particles, with average particle diameter of 130.9 ± 23.3 nm and 145.6 ± 23.1 nm for PTX@BMPH-MPMSN (Fig. 1a) and CHX-PTX@BMPH-MPMSN material (Fig. 1b), respectively. Statistical analysis revealed no significant difference in the diameter of these two materials (p > 0.05, n = 20). Transmission electron microscopy (TEM) images of the starting BMPH-MPMSN materials previously evidenced its mesoporous nature [34], with additional images shown in Fig. S1. The results of SEM-EDX analyses of the synthesized drug loaded materials are shown in Figs. S2 and S3. Nitrogen sorption isotherm changes from the type IV isotherm observed from MPMSN and BMPH-MPMSN to type I isotherm for PTX@BMPH-MPMSN and type II isotherm for CHX-PTX@BMPH-MPMSN (Fig. 1c), which is typically observed for microporous and nonporous materials, respectively. These changes in the obtained isotherms can be ascribed to surface modification, loading and capping the mesopores. Notably, the decrease in specific surface area (SSA) and total pore volume (TPV) was noted in case of modification of MPMSN into BMPH-MPMSN (Table 1), though both materials showed average pore size of 2.4 nm [34]. Furthermore, both SSA and TPV of novel PTX-loaded materials are substantially lower (Table 1), as expected after loading and capping the mesopores. The FTIR spectra confirm the structure of the obtained materials (Fig. 1d). Namely, wide stretching (O-H) vibrations are noted in the region 3000–3600 cm^−1^ and δ(O-H) centered at 1635 cm^−1^, which are ascribed to the adsorbed water and surface silanols. The stretching vibrations from the surface functionalized groups in all materials are observed at ca. 2924 and 2853 cm^−1^ (*ν*(C-H)), 1461 and 1403 cm^−1^ (*ν*(C-C)), 796 cm^−1^ (*ν*(Si-C)) and 684 cm^−1^ (*ν*(C-S)). In case of stretching Si-O vibrations for MPMSN and BMPH-MPMSN, the band ascribed to (*ν*(Si-OH)) is observed at 966 cm^−1^ while the broad band ascribed to *ν*(Si-O-Si) and *ν*(Si-O-C) is noted at 1077 cm^−1^. Additionally, in the case of BMPH-MPMSN the band at 1701 cm^−1^ is ascribed to the stretching vibrations of the carbonyl groups of BMPH [34]. The presence of PTX in the drug loaded materials is clearly observed by the occurrence of a broad band of increased intensity at 1702 cm^−1^, which can be ascribed to the stretching vibrations of the four ester and one keto groups on PTX. In comparison to the starting BMPH-MPMSN, the additional peak can be observed at 1493 cm^−1^ in the spectrum of PTX@BMPH-MPMSN and at 1495 cm^−1^ for CHX-PTX@BMPH-MPMSN which can be ascribed to the stretching vibration of the hydrazone linkages between the CD and BMPH, as well as the contribution of stretching bands from imine moieties which formed in reaction of GA with the amine groups from the AA and CHX, in case of CHX-PTX@BMPH-MPMSN. Similarly to our previous observation for the BMPH-MPMSN loaded with Gadobutrol [34], the shifting to lower energy of vibration of *ν*(Si-O-Si) bands occurs due to the presence of PTX on the surface and inside the mesopores, which slightly weakens the nearby Si-O bonds though hydrogen bonding with the silica surface. Thus, the vibration band that occurs at 1077 cm^−1^ in the spectrum of BMPH-MPMSN shifts to 1052 cm ^−1^ and 1047 cm^−1^ for PTX@BMPH-MPMSN and CHX-PTX@BMPH-MPMSN, respectively.

**Table 1:** BET surface area, total pore volume, loading capacity and DLS diameter of the prepared materials.

Material	BET surface area (m^2^/g±SD)	Total Pore Volume (cm^3^/g±SD)	Amount of loaded drug (mg PTX/g material±SD)	Hydrodynamic diameter (nm±SD)	Zeta potential (mA±SD)
MPMSN^a^	860 ± 20	0.53 ± 0.01	/	250 ± 10	−24 ± 1
BMPH-MPMSN^a^	770 ± 20	0.47 ± 0.01	/	250 ± 10	−27 ± 1
PTX@BMPH-MPMSN	234 ± 5	0.32 ± 0.01	17.2 ± 0.1	310 ± 60	−33 ± 1
CHX-PTX@BMPH-MPMSN	22 ± 1	0.17 ± 0.01	79.2 ± 0.2	370 ± 20	−40 ± 1

a Reproduced with permission from [34].

The results of TGA analysis show similar weight loss patterns for all materials in the employed conditions (Fig. S4a). The first derivative of the obtained results (dm/dT) reveals that there is one main degradation band in case of MPMSN with the peak at 347 °C (Fig. S4b). In case of BMPH-MPMSN, the first degradation peak shifts to 344 °C. while additional degradation appears, centered at ca. 600 °C, which can be ascribed to the functionalized BMPH. In case of PTX loaded materials, the first degradation peak shifts to 340 °C and shows the presence of the additional degradation peak at ca. 500 °C. Further evidence of the presence of capping molecules and loaded PTX within the mesopores is evidenced by the exothermic change above 540 °C in the DSC spectra of the PTX loaded materials, which is not observed in non-loaded materials (Fig. S4c). Table 1 gives information about the amounts of encapsulated PTX in the materials with and without the addition of targeting CHX ligands. These values were determined from the filtrates and the standard curves obtained with UV/VIS spectroscopy for PTX (Fig. S5), as detailed in the experimental section. Evidently, a substantially higher amount of PTX was loaded in the material that contained attached CHX, which might indicate increased capping efficacy upon binding the oligopeptide and isolating CHX-PTX@BMPH-MPMSN. The hydrodynamic diameters and of the prepared materials were measured in PBS and the obtained values were 310 ± 60 nm for PTX@BMPH-MPMSN and 370 ± 20 nm for CHX-PTX@ BMPH-MPMSN (Table 1). Zeta potential values for the obtained materials are also provided in Table 1 and distribution of zeta potential is shown on Fig. S6.

**Fig. 2:**
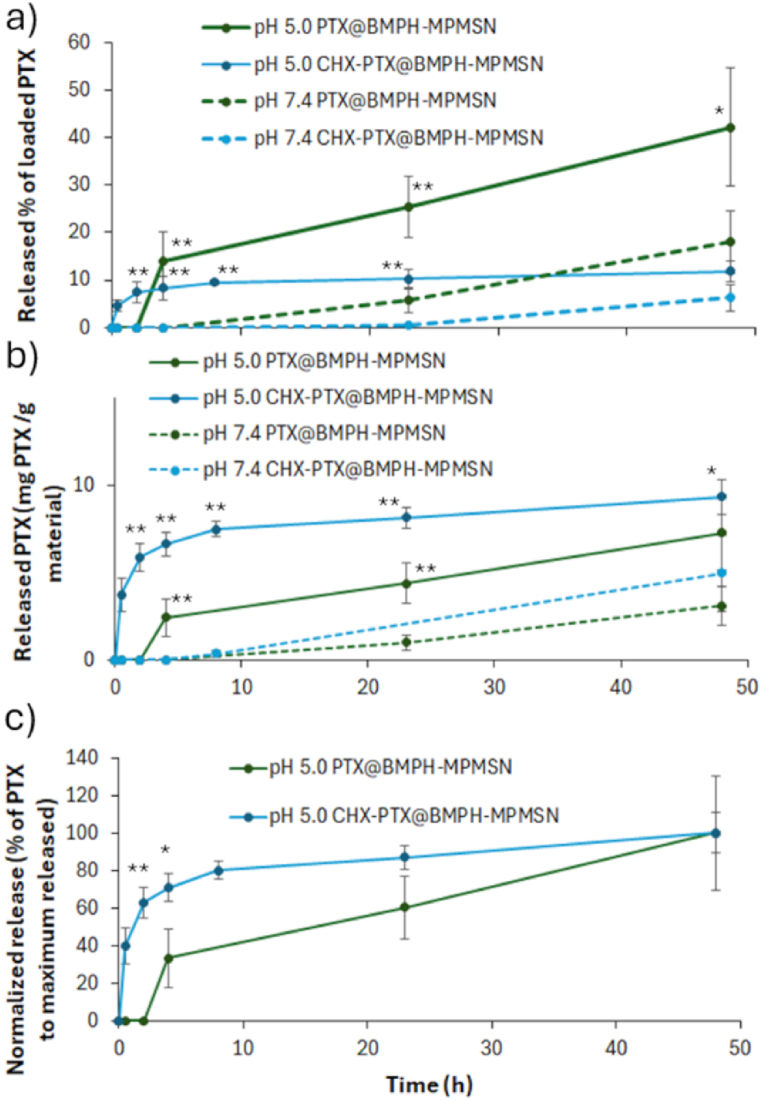
a) Release portion of the loaded PTX and b) the amount of released PTX/g of loaded material, at pH 5 and pH 7.4 from PTX@BMPH-MPMSN and CHX-PTX@BMPH-MPMSN; c) Normalized release kinetics of CHX-PTX@BMPH-MPMSN and PTX@BMPH-MPMSN at pH 5.0 (∗p < 0.05, ∗∗p < 0.01).

The release kinetics of PTX from the PTX@BMPH-MPMSN and CHX-PTX@BMPH-MPMSN materials was measured by UV/VIS measurements in the buffer (acetate pH 5.0 or PBS pH 7.4)/methanol mixtures (Fig. 2a,). Evidently, in the case of both materials, a significantly higher amount of PTX was released at pH 5.0 vs. pH 7.4. After 24 h, the released portion of the loaded PTX was under 5 % for both PTX@BMPH-MPMSN and CHX-PTX@BMPH-MPMSN at pH 7.4, demonstrating good pore-sealing effects of the capping moieties. On the other hand, in weakly acidic conditions (pH 5.0), the results showed that after 48 h 11.8 % of the loaded drug was released from CHX-PTX@BMPH-MPMSN and 42.2 % from the PTX@BMPH-MPMSN. Again, the presence of CHX seems to enhance the stability of the capping, which leads to a lower portion of the released PTX in the measured conditions. Nevertheless, the total amounts of PTX released after 48 h of stirring at pH 5.0 was higher in case of PTX/g of CHX-PTX@BMPH-MPMSN (9.3 mg PTX/g of material) than in the case of PTX/g of PTX@BMPH-MPMSN (7.3 mg PTX/g of material) as shown in Fig. 2b. This result is clearly a consequence of higher PTX loading in CHX-PTX@BMPH-MPMSN. However, based on the graphs it is evident that the release of PTX from CHX-PTX@BMPH-MPMSN followed much faster kinetics, with 80 % of released PTX achieved after 4h, while only 33 % of PTX is released from PTX@BMPH-MPMSN at the same time point (Fig. 2c). This difference is important as faster kinetics of drug release could lead to enhanced toxicity as cells get early exposure to higher local drug concentration. Gradual release of PTX was observed in both materials, which is typical for mesoporous silica-based drug carriers and can be described as a two-step process governed by Fickian diffusion from the mesopores [36]. Comparison of the measured spectra from the release kinetics measurements at pH 5 and different time points is provided in supplemental information (Fig. S7), which reveals that additional broad band appears in the supernatants from the CHX-functionalized material in the range 250–290 nm, which can be ascribed to the released CHX. As determination of PTX is conducted by measurements at 230 nm, while proteins and peptides primarily absorb UV light due to the presence of tryptophan, tyrosine, and phenylalanine residues, with absorbance maxima at 280, 275, and 258 nm, respectively [37], no significant influence on PTX determination is expected.

**Fig. 3:**
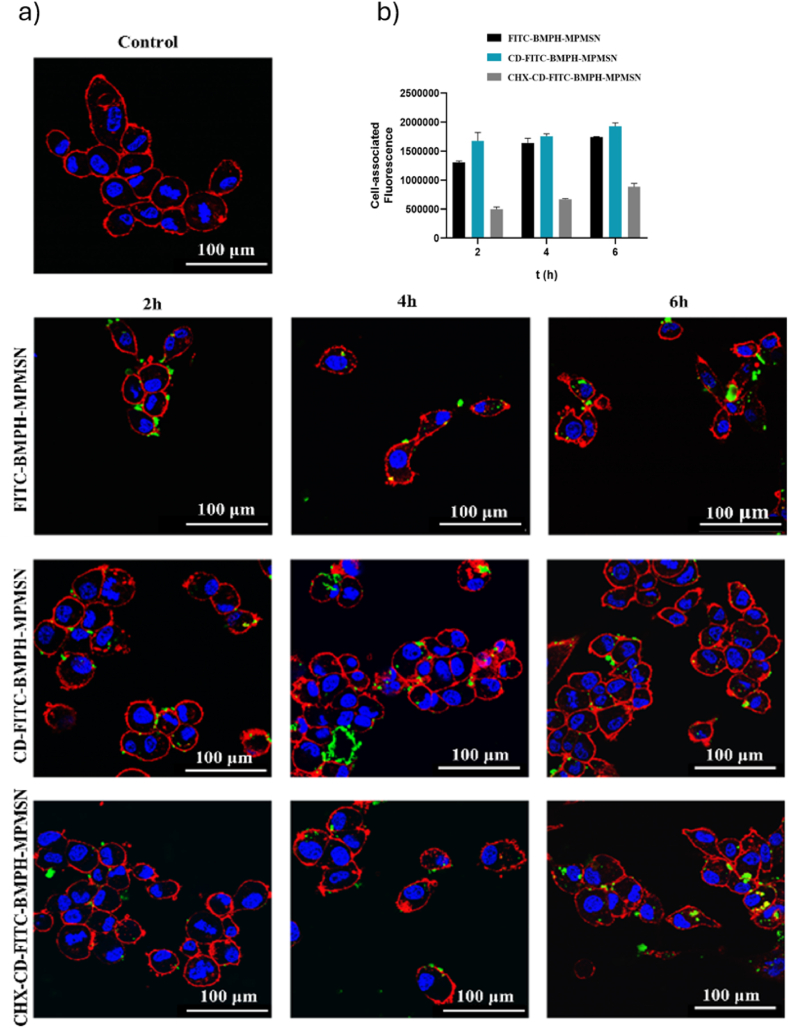
**a)** Representative confocal microscopy images of U87 cells after 2, 4 or 6 h of incubation with 50 μg/mL of FITC-BMPH-MPMSN, CD-FITC-BMPH-MPMSN or CHX-CD-FITC-BMPH-MPMSN. Cell nuclei are stained with Hoechst 33258 (blue) and cellular membrane with ActinRed™ 555 ReadyProbes™ Reagent (red). NPs are labeled with FITC (green). Scale bar = 100 μm. b) Cell-associated fluorescence over time of U87 cells treated with 50 μg/mL of FITC-BMPH-MPMSN, CD-FITC-BMPH-MPMSN or CHX-CD-FITC-BMPH-MPMSN, expressed as mean ± SD (n = 2). Two-way ANOVA and Sidak's multiple comparisons test were applied (p < 0.05).

To evaluate the potential for biological applications of these nanoparticles, we first investigated the internalization capability of the prepared materials in U87 cells, using confocal microscopy and flow cytometry. The confocal microscopy results at different time points are shown in Fig. 3a. We tested three different materials labeled with FITC without any cargo molecules: the first one with no capping, the second one capped with CD and the third one capped with CD and functionalized with CHX. All the materials showed the ability to enter U87 cells after 4 or 6 h. The results are in line with our expectations, internalization of MSNs usually occurs a few hours after their administration, although there may be variations depending on the functionalization of the NPs or the employed cell line [[38], [39], [40]].

The flow cytometry results (Fig. 3b and Fig. S8) revealed that CD-FITC-BMPH-MPMSN are the best internalized NPs by U87 cell, while the uptake of CHX-CD-FITC-BMPH-MPMSN is lower than the other two. This result could be explained by the possible agglomeration in the cell medium of the NPs after functionalization with CHX, which then hinders their endocytosis.

To prove this assumption, DLS measurements were performed on the suspension of the capped materials without loaded cargo molecules in cell medium at different time points (Fig. S9 and S10). The measurements revealed a hydrodynamic diameter of 584 ± 77 nm for CD-BMPH-MPMSN and 739 ± 58 nm for CHX-CD-BMPH-MPMSN immediately after preparing the dispersion and intensive sonication, that increased to 808 ± 81 nm and 980 ± 181 nm, respectively, after 1h incubation. Therefore, both types of particles show a tendency to agglomerate in cell medium, with the enhanced agglomeration for the material functionalized with CHX. The agglomeration of particles in PBS is less pronounced, evidenced by the hydrodynamic diameter of PTX@BMPH-MPMSN measured as 311 ± 55 nm before and 334 ± 83 nm after 1h incubation in PBS, while for CHX-PTX@ BMPH-MPMSN, the obtained values were 374 ± 15 nm before and 503 ± 42 nm after 1 h incubation (Fig. S11). However, even in PBS the CHX-functionalized material shows enhanced susceptibility to agglomeration vs. the material without CHX, as clearly evidenced by the shift of the peaks to higher diameters after 1 h of incubation.

**Fig. 4:**
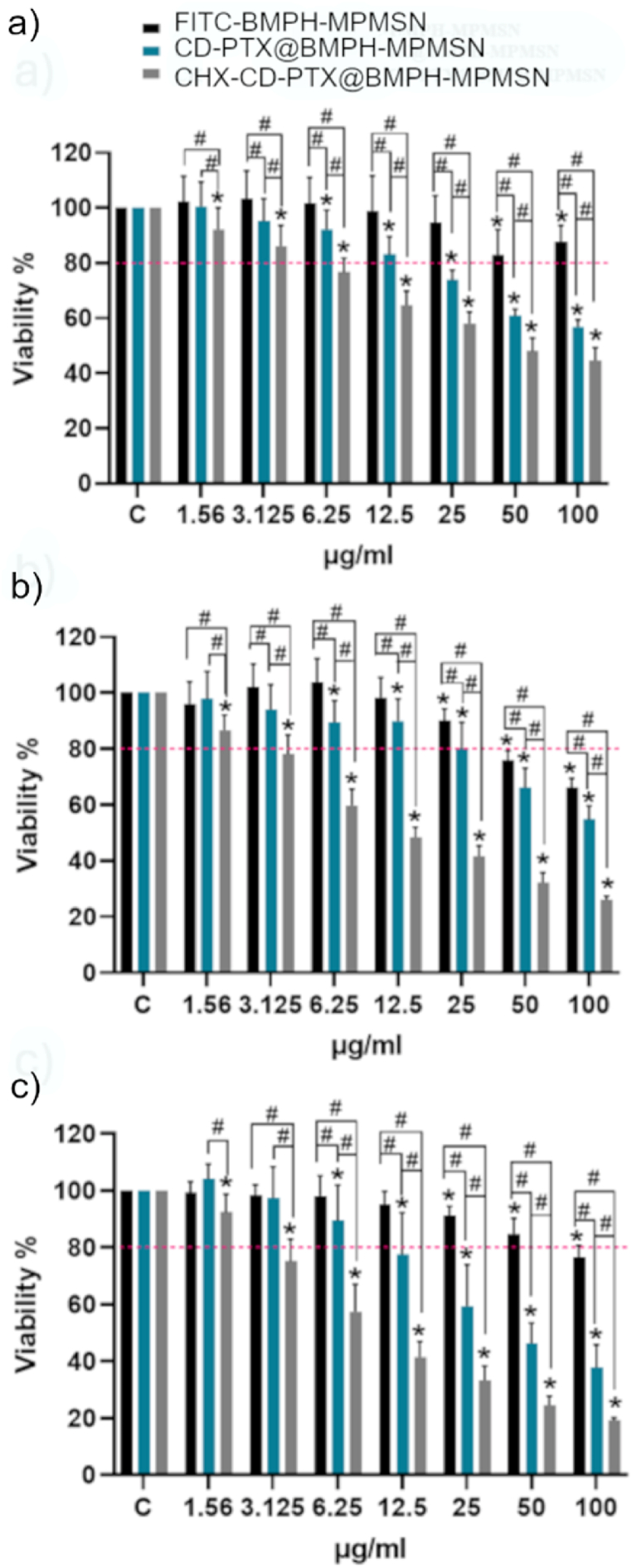
U87 cells viability after the incubation with different concentrations of FITC-BMPH-MPMSN, PTX@BMPH-MPMSN or CHX-PTX@BMPH-MPMSN for a) 24 h, b) 48 h and c) 72 h. Cell viability was analyzed by measuring absorbance at 450 nm with the CCK-8 reagent. Data are expressed as mean ± SD. Two-way ANOVA and Sidak's multiple comparisons test were applied (∗p < 0.05 vs control, ^#^p < 0.05).

The cytotoxic effects of the materials loaded with PTX were further evaluated on the same cell line. As can be observed in Fig. 4, FITC-labeled starting material (FITC-BMPH-MPMSN) did not show significant toxicity except at a concentration of 100 μg/mL, after 48 or 72 h of exposure. The corresponding FITC-labeled materials without loaded cargo but capped only with CD, or with CD and CHX, do not appear to affect cell viability even at the highest concentrations and longer exposure times tested (Fig. S12). However, upon co-incubating the cells with materials loaded with PTX, a significant enhancement of the toxicity was noted even after 24h of treatment. As the experiments were performed in media at physiological pH, we presume that the acidic environment of the endosomes is triggering the release of the cargo loaded from the NPs. A notable difference was found also between the two materials loaded with PTX. Evidently, the NPs with CHX on the surface induced a higher cytotoxic effect on U87 cells, despite the previously observed lower cellular uptake, which agrees with a higher loaded amount of PTX in the CHX-capped material.

## 4 Conclusion

The anticancer drug PTX was successfully entrapped inside pH-responsive mesoporous silica-based nanomaterials. The PTX-loaded MSNs were capped with β-cyclodextrin monoaldehyde, covalently attached to the hydrazide-functionalized MSN, and further functionalized with CHX as a potential targeting ligand for glioblastoma multiforme. The release of the anti-cancer drug PTX was increased in the weakly acidic condition compared to the release at physiological pH. Confocal microscopy and flow cytometry measurements revealed that all tested materials are endocytosed by the U87 cells. Furthermore, it was demonstrated that PTX-loaded materials exhibit a cytotoxic effect on these cells, with the highest toxicity observed for the CHX-functionalized PTX-loaded material. This result is promising toward possible applications in controlled delivery of anticancer drugs to Glioblastoma multiforme tissues in response to their acidic environment, with the enhanced activity upon functionalization with CHX.

## CRediT authorship contribution statement

**Mirjana Mundžić:** Writing – original draft, Visualization, Validation, Methodology, Investigation, Formal analysis. **Amelia Ultimo:** Writing – review & editing, Visualization, Validation, Methodology, Investigation, Formal analysis, Conceptualization. **Minja Mladenović:** Writing – review & editing, Visualization, Validation, Investigation, Formal analysis. **Aleksandra Pavlović:** Writing – review & editing, Visualization, Investigation, Formal analysis. **Oliviero L. Gobbo:** Writing – review & editing, Supervision, Resources, Methodology. **Eduardo Ruiz-Hernandez:** Writing – review & editing, Project administration, Funding acquisition, Conceptualization. **Maria Jose Santos-Martinez:** Writing – review & editing, Supervision, Project administration, Methodology. **Nikola Ž. Knežević:** Writing – review & editing, Writing – original draft, Validation, Supervision, Project administration, Methodology, Funding acquisition, Conceptualization.

## Data availability statement

Data will be made available on request.

## Funding sources

This work was supported by the Science Fund of the Republic of Serbia, PROMIS, #6060755, PRECAST, the European Union's Horizon 2020 research and innovation programme under grant agreement 952259 (NANOFACTS), Horizon 2020 Teaming program ANTARES (grant No. 664387) and the European Research Council under grant agreement 758887 (REACT).

## Declaration of competing interest

The authors declare that they have no known competing financial interests or personal relationships that could have appeared to influence the work reported in this paper.
