# Supplementary material for: Chlorotoxin-functionalized mesoporous silica nanoparticles for pH-responsive paclitaxel delivery to Glioblastoma multiforme
Source: Heliyon. 2024 Dec 14;11(1):e41151. doi: 10.1016/j.heliyon.2024.e41151 (PMC11699378; doi:10.1016/j.heliyon.2024.e41151)
Supplement: Multimedia component 1 [file mmc1.docx]

*Supplementary*

**Chlorotoxin-functionalized Mesoporous Silica Nanoparticles for pH-responsive Paclitaxel Delivery to Glioblastoma Multiforme**

*Mirjana Mundžić, ^a^ Amelia Ultimo, ^b^ Minja Mladenović, ^a^ Aleksandra Pavlović, ^b^ Oliviero L. Gobbo, ^b, c^ Eduardo Ruiz-Hernandez, ^b, c^ Maria Jose Santos-Martinez ^b, c, d^ and Nikola Ž. Knežević ^a^**

^a^BioSense Institute, University of Novi Sad, Dr Zorana Djindjica 1, 21000 Novi Sad, Serbia

^b^School of Pharmacy and Pharmaceutical Sciences, Panoz Institute, Trinity College Dublin, D02PN40, Dublin, Ireland

^c^Trinity St. James’s Cancer Institute, St James’s Hospital, D08 NHY1, Dublin, Ireland

^d^School of Medicine, Trinity College Dublin, D02 E8C0 Dublin, Ireland

*****Corresponding author: [nknezevic@biosense.rs](mailto:nknezevic@biosense.rs)


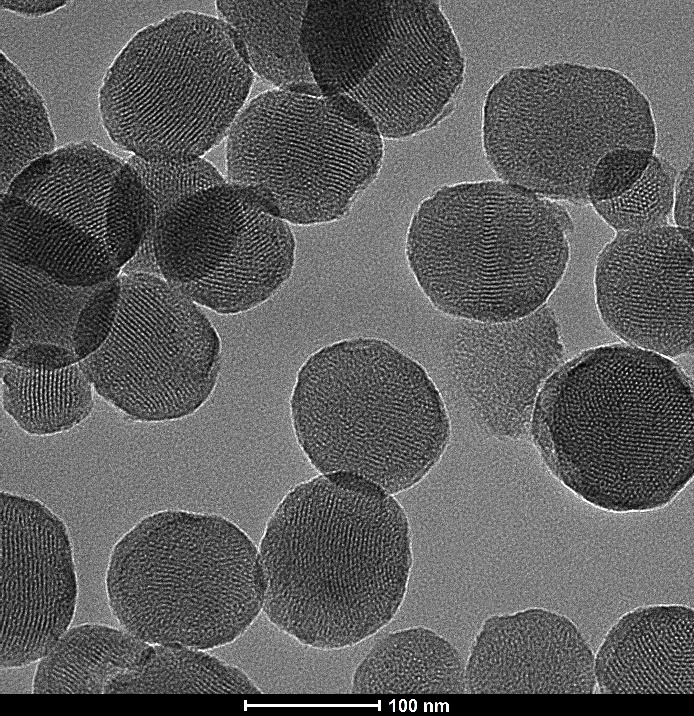

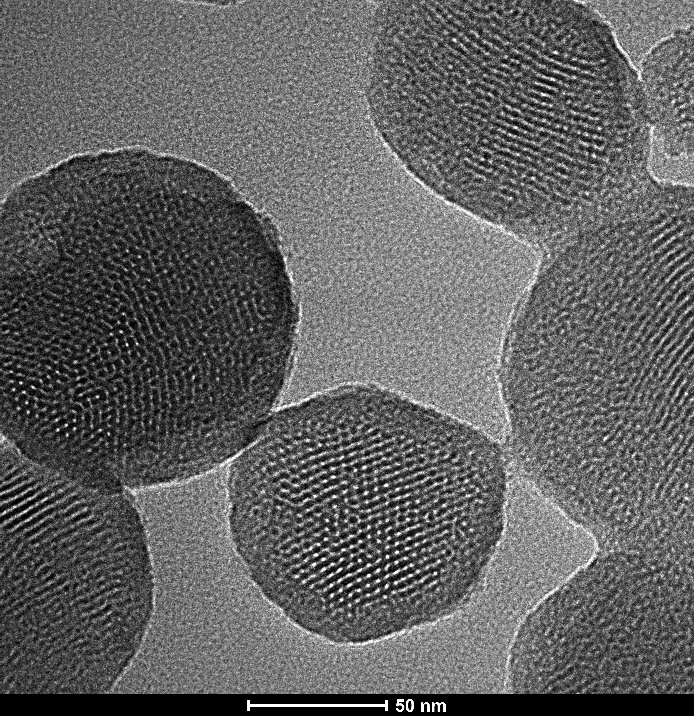

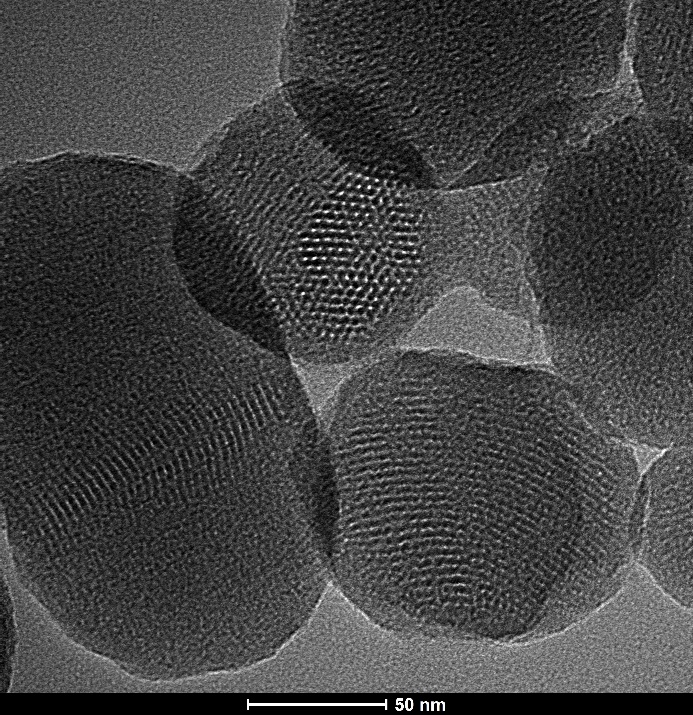


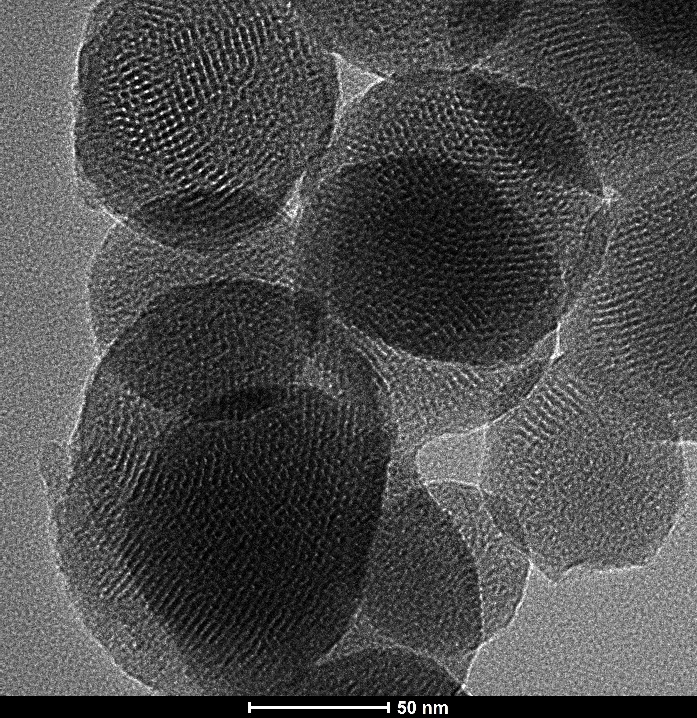


**Figure S1.** TEM images of BMPH-MPMSN.


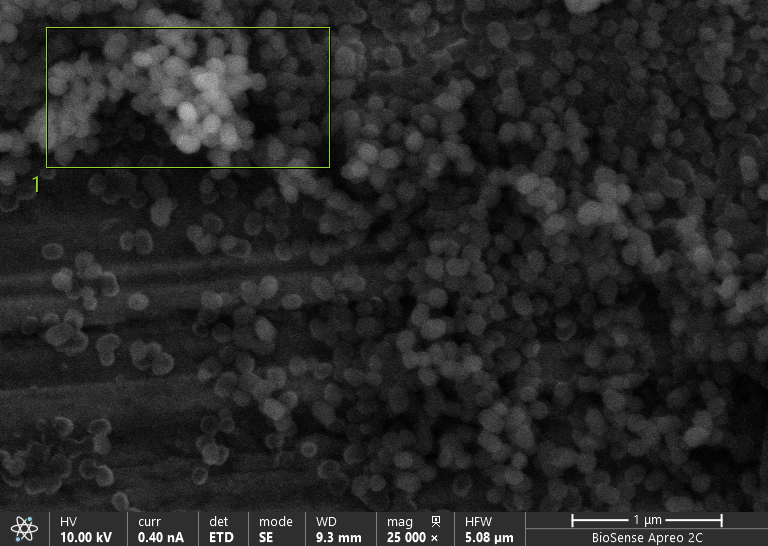


Total Number of Counts: 65 556
Average Count Rate: 3 859 cps
Acceleration Voltage: 10 kV
Total Acquisition Time: 17 seconds

| **Element** | **Line** | **At. %** | **Wt. %** | **Net Counts** | **At. % Error** | **Wt. % Error** |
| --- | --- | --- | --- | --- | --- | --- |
| **C** | **K** | **37.4** | **27.3** | **1 522** | **1.1** | **0.8** |
| **O** | **K** | **47.5** | **46.3** | **2 264** | **1.6** | **1.6** |
| **Si** | **K** | **12.6** | **21.6** | **2 192** | **0.5** | **0.8** |
| **S** | **K** | **2.5** | **4.8** | **402** | **0.3** | **0.5** |

**
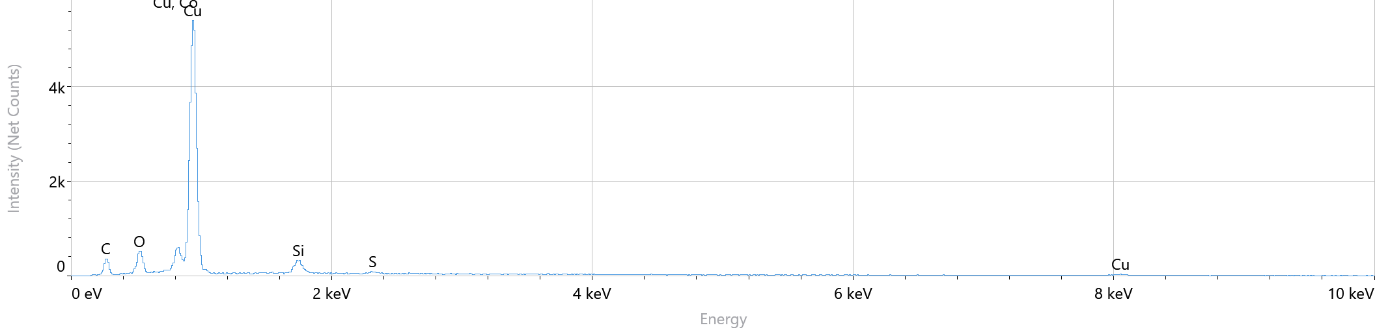
**

Figure S2. SEM-EDX analysis of PTX@MPH-MPMSN


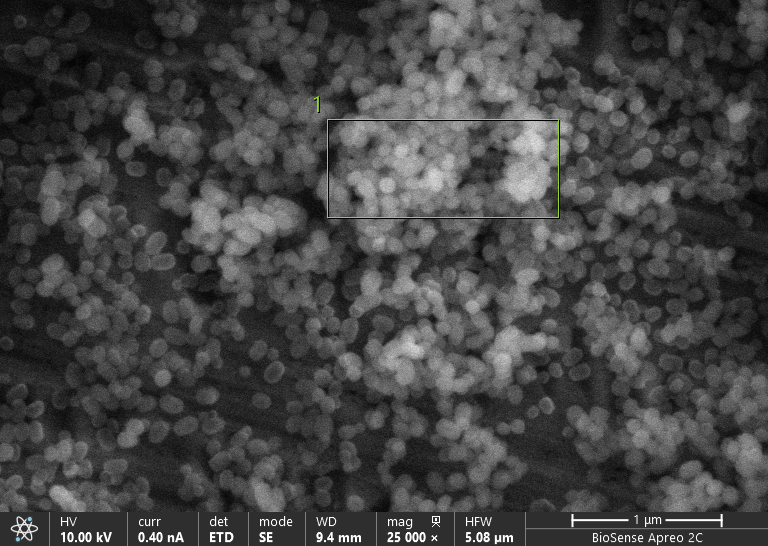


Total Number of Counts: 152 930
Average Count Rate: 2 549 cps
Acceleration Voltage: 10 kV
Total Acquisition Time: 60 seconds

| **Element** | **Line** | **At. %** | **Wt. %** | **Net Counts** | **At. % Error** | **Wt. % Error** |
| --- | --- | --- | --- | --- | --- | --- |
| **C** | **K** | **13.8** | **9.0** | **1 755** | **0.4** | **0.3** |
| **O** | **K** | **62.3** | **54.1** | **14 438** | **0.7** | **0.6** |
| **Si** | **K** | **22.0** | **33.5** | **15 386** | **0.2** | **0.4** |
| **S** | **K** | **1.9** | **3.4** | **1 224** | **0.1** | **0.2** |


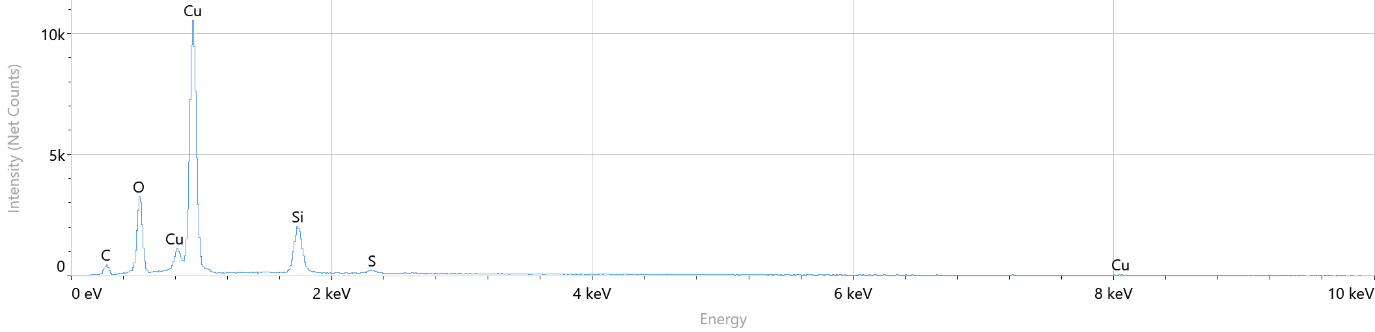
Figure S3. SEM-EDX analysis of CHX-PTX@MPH-MPMS.


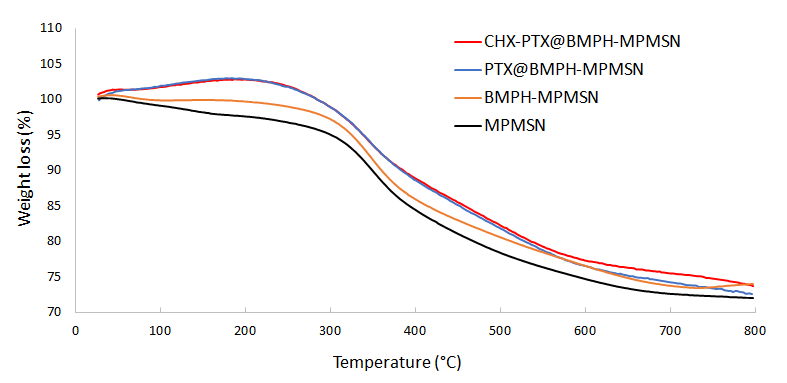


a)

b)


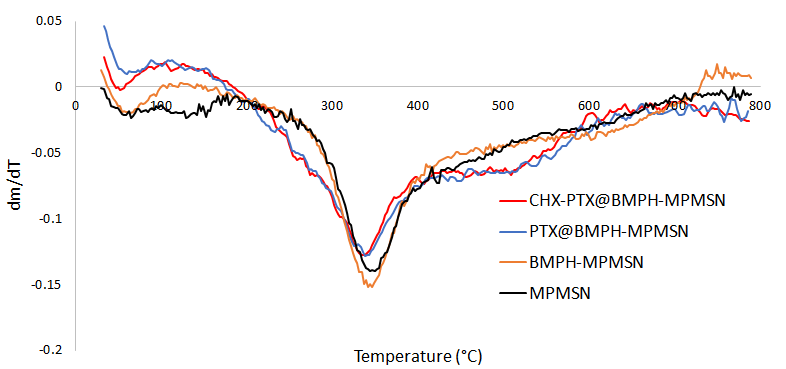


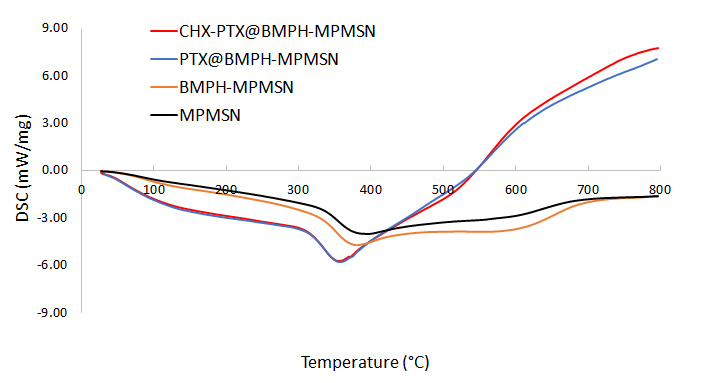


c)

**Figure S4.** a) The thermogravimetry (TGA); b) the first derivative dm/dT and c) differential scanning calorimetry (DSC) results for MPMSN, BMPH-MPMSN, PTX@BMPH-MPMSN and CHX-PTX@BMPH-MPMSN.

**
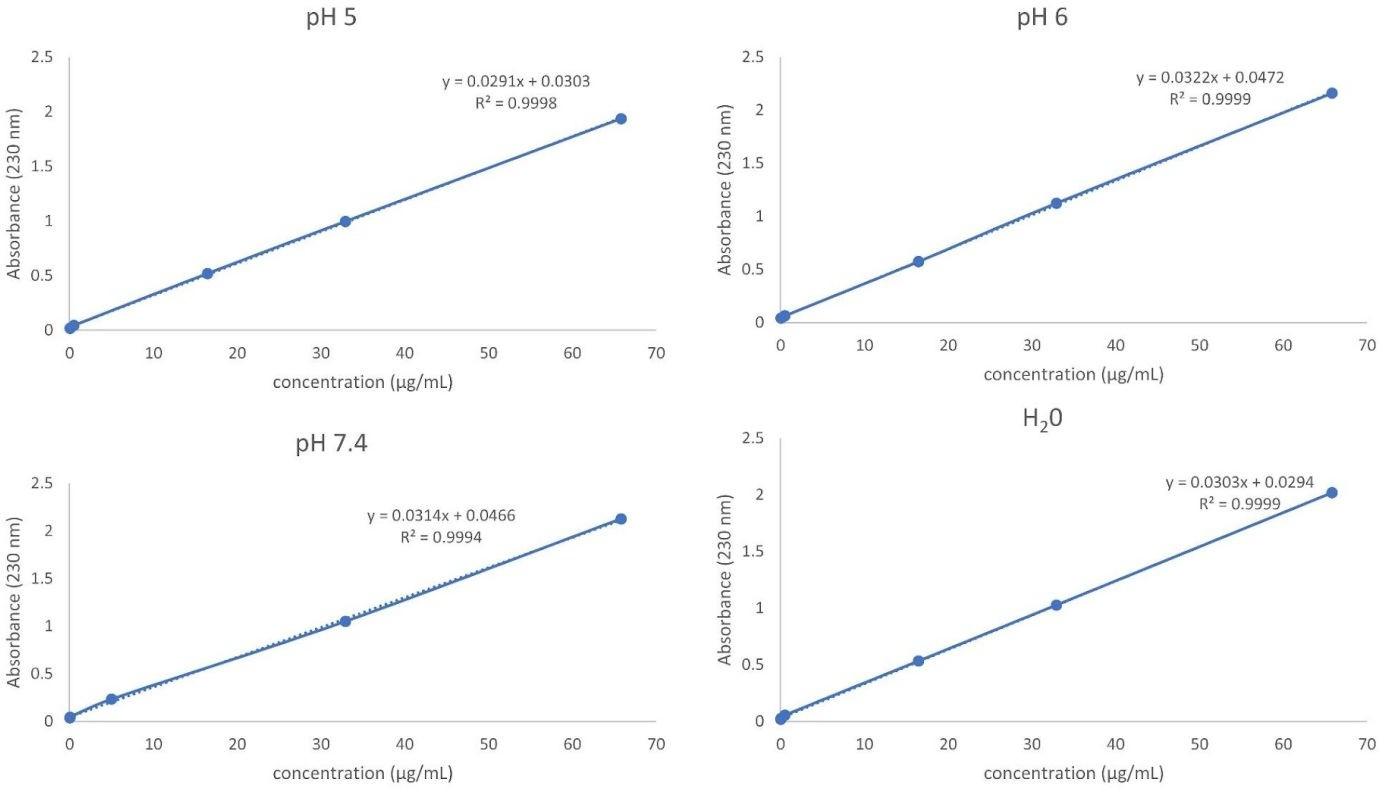
**

**Figure S5.** Calibration curves for Paclitaxel aqueous solution containing 65.8% of methanol (absorbance at 230 nm) at different pH.


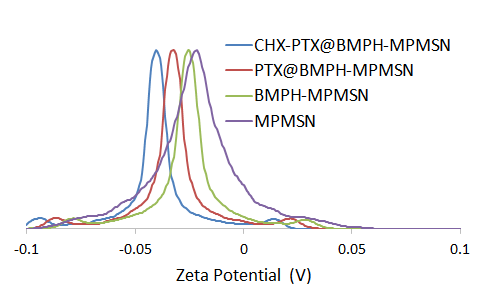


**Figure S6.** Zeta potential distribution for CHX-PTX@BMPH-MPMSN, PTX@BMPH-MPMSN, BMPH-MPMSN and MPMSN

a)

b)
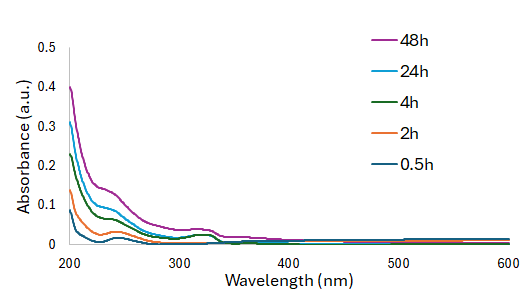


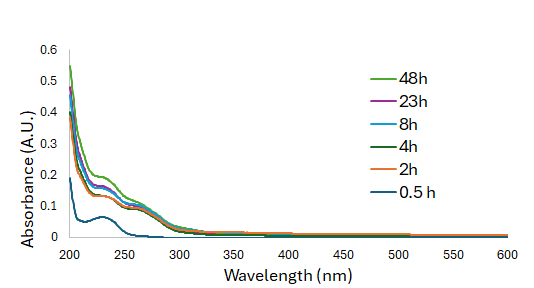


**Figure S7**. UV/VIS absorbance spectra of supernatants from stirred suspensions of a) PTX@BMPH-MPMSN and b) CHX-PTX@BMPH-MPMSN in pH 5.0 buffer at different time points


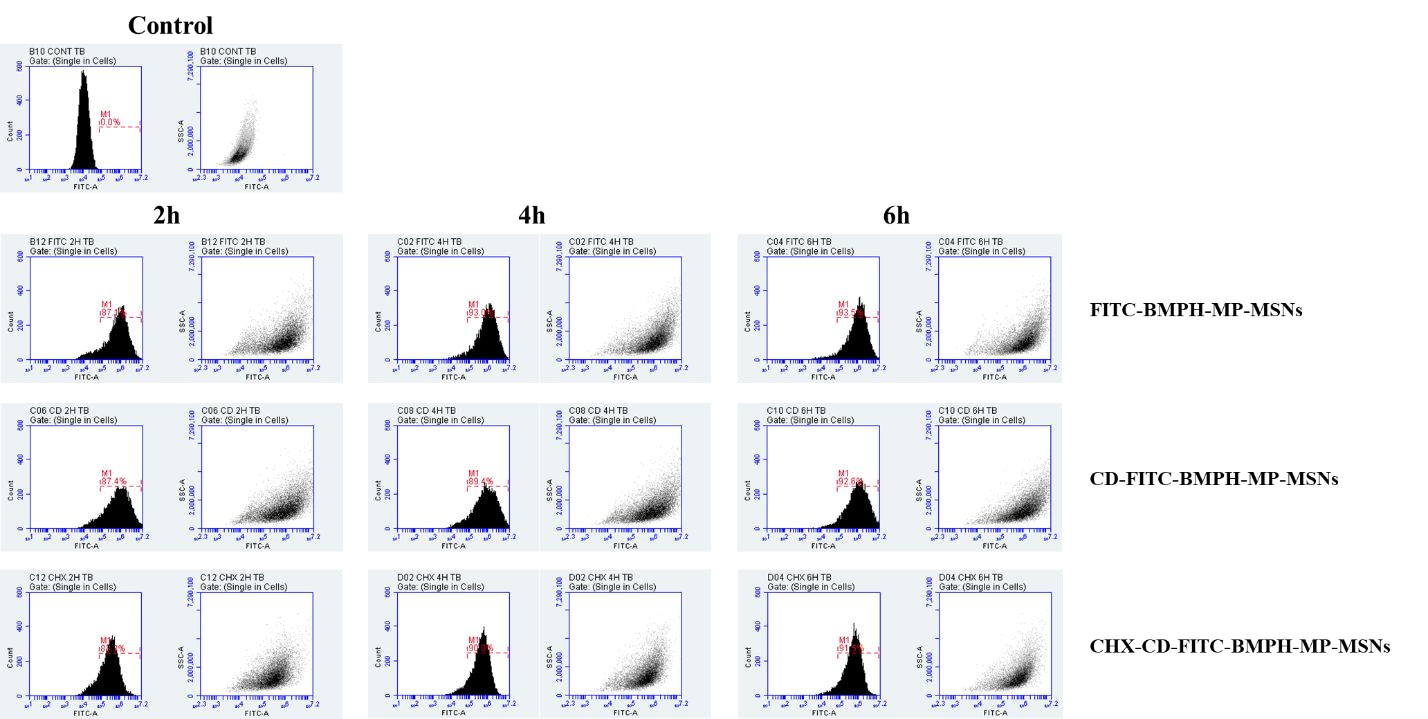


**Figure S8.** Representative histograms and dot plots showing FITC-BMPH-MP-MSNs, CD-FITC-BMPH-MPMSN and CHX-CD-FITC-BMPH-MPMSN internalization in U87 cells at different time points.


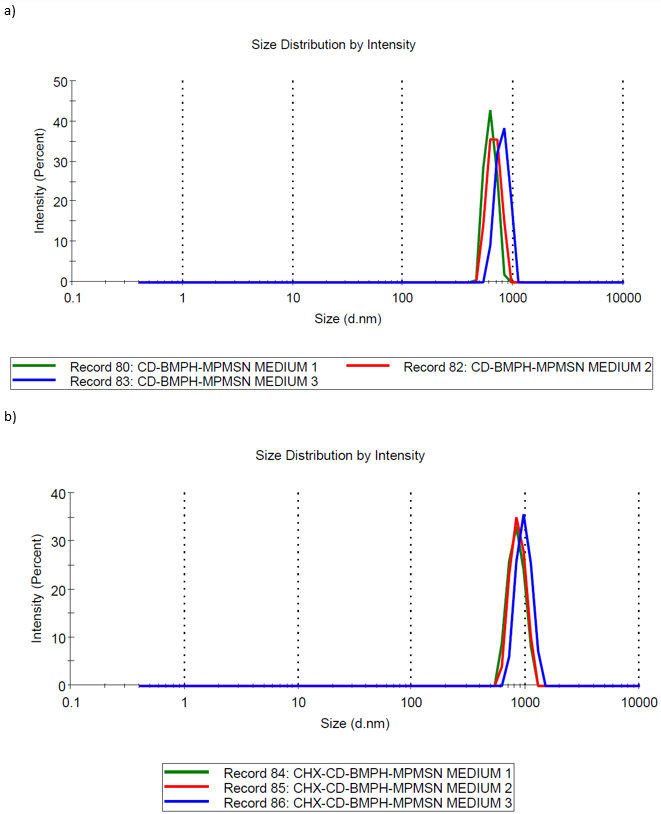


**Figure S9.** Triplicate DLS size measurements in cell medium at 0.1 mg/mL concentration immediately after dispersing: a) CD-BMPH-MPMSN Z-average = 840.8 nm, PdI = 0.456 and b) CDX-CD-BMPH-MPMSN, Z-average = 1026 nm, PdI = 0.075.


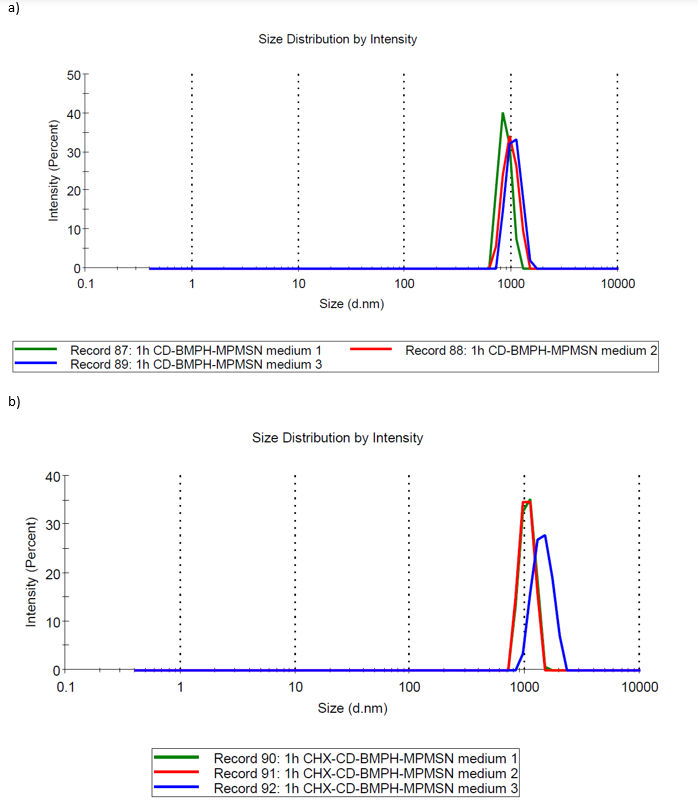


**Figure S10.** Triplicate DLS size measurements in cell medium at 0.1 mg/mL concentration 1h after dispersing: a) CD-BMPH-MPMSN, Z-average = 1086 nm, PdI = 0.414 and b) CDX-CD-BMPH-MPMSN, Z-average = 1197 nm, PdI = 0.140.


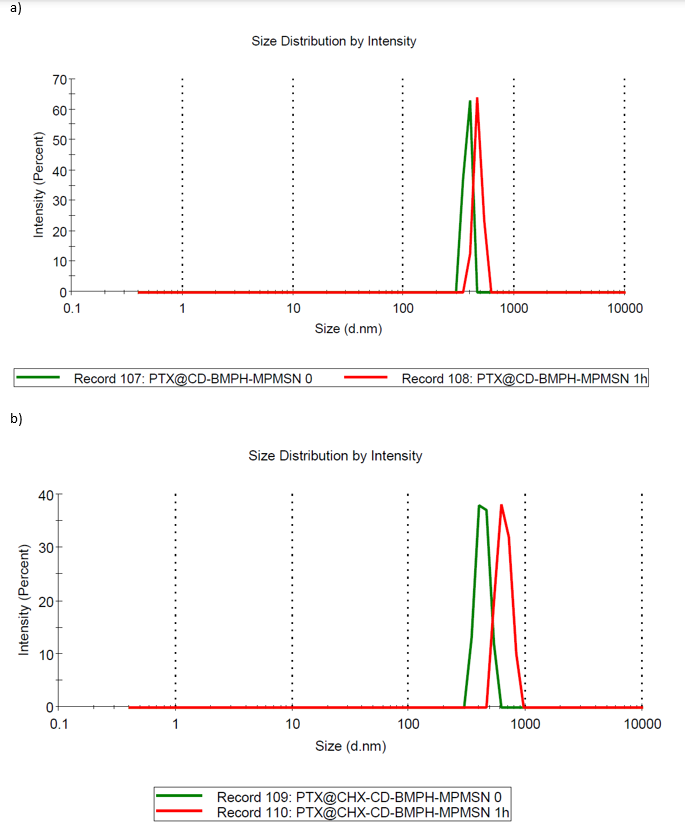


**Figure S11.** DLS size measurements in PBS at 0.05 mg/mL concentration immediately after dispersing and after 1h of incubation of: a) PTX@BMPH-MPMSN, PdI = 0.859 and b) CHX-PTX@BMPH-MPMSN, PdI = 0.584.

**
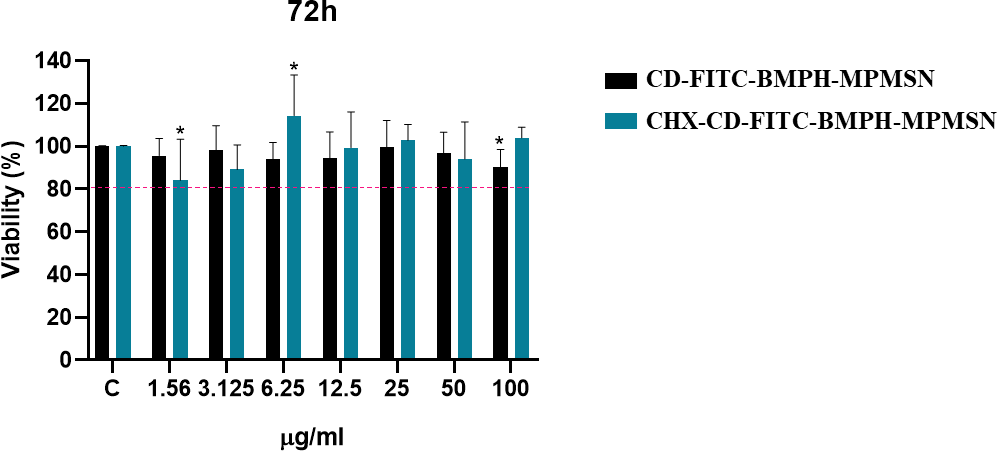
**

**Figure S12**. U87 cells viability after 72 h of incubation with different concentrations of CD-FITC-BMPH-MP- MSNs or CHX-CD-FITC-BMPH-MP-MSNs. Cell viability was analyzed by measuring absorbance at 450 nm with the CCK-8 reagent. Data are expressed as mean ± SD. Two-way ANOVA and Sidak’s multiple comparisons test were applied (* p < 0.05 vs control)
